# Development of artificial gravity and resistive vibration exercise protocol: A step towards novel countermeasures for prolonged spaceflight

**DOI:** 10.1113/EP093837

**Published:** 2026-05-29

**Authors:** Riley A. Dienna, Sabrina K. C. Ehrenfort, Cassandra M. Juran, Scott K. Ferguson

**Affiliations:** ^1^ Integrative Aerospace and Exercise Physiology Laboratory Department of Human Factors College of Arts and Sciences Embry‐Riddle Aeronautical University Daytona Beach Florida USA; ^2^ Space Physiology Antibody and Cellular Engineering Laboratory Department of Human Factors College of Arts and Sciences Embry‐Riddle Aeronautical University Daytona Beach Florida USA; ^3^ Blue Marble Space Institute of Science Seattle Washington USA

**Keywords:** aerospace physiology, bone degeneration, microgravity, space flight

1

Prolonged exposure to microgravity induces a distinct set of physiological adaptations that often become most apparent upon return to Earth's gravity (1 G). Among these, astronauts completing extended missions aboard the International Space Station commonly exhibit orthostatic intolerance, elevations in intraocular pressure and musculoskeletal deconditioning. This collection of maladaptive physiological changes is referred to as spaceflight‐induced physiological deconditioning (Demontis et al., 2017) and substantially reduces aerobic capacity and performance in astronauts. The health risks posed by exposure to microgravity are especially concerning as plans are made for extended lunar missions and the 9 month journey to Mars, and these complications could endanger the crew during and after the mission. Since our early sojourns into space, a variety of exercise countermeasures have been implemented to mitigate spaceflight‐induced physiological deconditioning, including Exerciser 1 during the 1974–1975 Skylab missions and the Advanced Resistance Exercise Device aboard the International Space Station. Despite best efforts, these countermeasures do not fully mitigate microgravity‐induced maladaptation, particularly the progressive loss of skeletal muscle and bone mass and the deterioration of bone microarchitecture, which continues over long‐duration missions (Axpe et al., 2020).

One challenge in developing effective countermeasures is creating an analogue that accurately simulates on Earth the conditions experienced by astronauts in microgravity. Head‐down tilt bed rest (HDTBR) is often used to simulate the fluid shifts, muscular degeneration and bone resorption associated with prolonged mechanical unloading and serves as an analogue for long‐duration exposure to microgravity. Resistive vibration exercise on a horizontal platform during HDTBR has been shown to attenuate some aspects of musculoskeletal deconditioning. This includes enhancement of osteogenic signalling through activation of osteocyte mechanotransduction via increased fluid micro‐accelerations in the lacunar–canalicular network, reduction of recruitment of bone‐resorbing progenitor cells to the endosteal surface, and preservation of muscle mass through low‐frequency vibration‐induced reflexes that recruit motor units and maintain neuromuscular activation despite unloading.

In this issue of *Experimental Physiology*, Fortune et al. (2026) aimed to build upon current countermeasures by performing the 60‐day Bed Rest with Artificial Gravity and Resistive Vibration Exercise (BRAVE) study. Artificial gravity is an emerging countermeasure that leverages a short‐arm human centrifuge (SAHC) to load the body in a manner consistent with Earth‐normal gravitational stress; however, further research is required to establish its physiological effectiveness and operational feasibility as a long‐duration countermeasure. This investigation evaluated the use of SAHC and resistive vibration exercise protocols to determine the tolerance of participants to the training regimen during artificial gravity and HDTBR and to provide recommendations for future research. The primary objectives were to describe and evaluate artificial gravity resistive vibration exercise (AGRVE) and horizontal resistive vibration exercise (HRVE) protocols as countermeasures for physiological deconditioning associated with microgravity. A 60 day, 6° hypoxic HDTBR model was used to simulate both mechanical unloading and reduced oxygen conditions associated with spaceflight. Success of the protocol was evaluated through feasibility metrics, including session adherence, incidence of motion sickness and delayed‐onset muscle soreness. Building on previous work combining cycle ergometry with prolonged bed rest and artificial gravity, the AGRVE and HRVE protocols were refined to test the efficacy of resistive vibration exercise in both centrifugal and horizontal environments (Hedge et al., 2025). The primary difference between the two protocols lies in orientation and gravitational loading. The HRVE protocol uses a horizontal orientation to reduce orthograde loading stress, whereas the AGRVE protocol is conducted on a horizontally aligned SAHC, whereby angular velocity generates a head‐to‐foot artificial gravitational vector.

Fortune et al. (2026) suggest that both groups demonstrated high training adherence, with HRVE showing slightly greater adherence compared with AGRVE. Early interruptions to AGRVE were caused primarily by motion sickness in the early stages of the study. In the HRVE group, training intensity was reduced owing to fatigue and discomfort, whereas adjustments in AGRVE sessions were implemented to mitigate presyncope symptoms. Motion sickness occurred primarily during the first 4 weeks of training, with the introduction of frequent AGRVE sessions, and diminished following vestibular habituation. Although low levels of delayed‐onset muscle soreness were reported for both protocols owing to the submaximal loading strategy, late‐stage soreness occurred more frequently in the HRVE group, probably owing to constant bidirectional resistance. Both groups demonstrated similar increases in maximal strength, with early gains primarily attributable to neuromuscular adaptations.

Another valuable aspect of the study is its critical evaluation of the training protocols. Fortune et al. (2026) identify that the AGRVE protocol could be improved through a more gradual familiarization period, which might enhance adherence and reduce motion sickness during initial sessions. They also suggest that minimizing head movement during SAHC exposure might mitigate motion sickness, thereby improving tolerability and overall training compliance. These recommendations provide practical guidance for refining artificial gravity‐based countermeasures for both spaceflight applications and ground‐based analogues, such as prolonged bed rest. In parallel, recent work has explored the use of SAHC‐generated artificial gravity to maintain physiological function, particularly orthostatic tolerance, during periods of mechanical unloading (Kourtidou‐Papadeli et al., 2021). The protocols developed by Fortune et al. (2026) contribute to this growing body of research and might inform future clinical and operational applications.

Development of effective countermeasures to mitigate spaceflight‐induced deconditioning remains a significant challenge, owing to the unique physiological demands of microgravity. The BRAVE study represents an important step towards establishing the feasibility and implementation of artificial gravity‐based countermeasures during prolonged unloading. Although the findings provide valuable operational insight, they also highlight the need for continued research to determine the long‐term efficacy of artificial gravity. As human space exploration extends to longer‐duration missions, the development of safe and effective strategies to preserve the health of astronauts will be essential. Moreover, advances in space physiology research continue to inform approaches to maintaining health during prolonged inactivity on Earth, reinforcing the broader relevance of this work.

## AUTHOR CONTRIBUTIONS

All authors have read and approved the final version of this manuscript and agree to be accountable for all aspects of the work. All persons designated as authors qualify for authorship, and all those who qualify for authorship are listed.

## CONFLICT OF INTEREST

None declared.

## References

[eph70326-bib-0001] Axpe, E. , Chan, D. , Abegaz, M. F. , Schreurs, A. S. , Alwood, J. S. , Globus, R. K. , & Appel, E. A. (2020). A human mission to Mars: Predicting the bone mineral density loss of astronauts. PLoS ONE, 15(1), e0226434. 31967993 10.1371/journal.pone.0226434 PMC6975633

[eph70326-bib-0002] Demontis, G. C. , Germani, M. M. , Caiani, E. G. , Barravecchia, I. , Passino, C. , & Angeloni, D. (2017). Human pathophysiological adaptations to the space environment. Frontiers in Physiology, 8, 547. 28824446 10.3389/fphys.2017.00547 PMC5539130

[eph70326-bib-0003] Fortune, J. , Riccardo, G. , Sorrentino, S. Z. , Victorien, F.‐R. , Lydia, T. , Jason, T. F. , Sara, P. , Leonidas, G. I. , Ursa, C. , Igor, B. M. , & Adam, C. M. (2026). Development of and adherence to artificial gravity and resistive vibration exercises during 60 days of hypoxic 6° head‐down tilt bed rest: BRAVE study. Experimental Physiology, 111(7), 3152–3172. 10.1113/EP093699 PMC13327306 42207668

[eph70326-bib-0004] Hedge, E. T. , Bryans, C. G. , Mastrandrea, C. J. , Siebenmann, C. , Hargens, A. R. , Karlsson, L. L. , Hughson, R. L. , & Linnarsson, D. (2025). Exercise during artificial gravity preserves cardiorespiratory fitness but not orthostatic tolerance following 60 days of head‐down bed rest (BRACE). Journal of Applied Physiology, 139(4), 943–950. 40953072 10.1152/japplphysiol.00224.2025

[eph70326-bib-0005] Kourtidou‐Papadeli, C. , Frantzidis, C. A. , Gilou, S. , Plomariti, C. E. , Nday, C. M. , Karnaras, D. , Bakas, L. , Bamidis, P. D. , & Vernikos, J. (2021). Gravity threshold and dose‐response relationships: Health benefits using a short‐arm human centrifuge. Frontiers in Physiology, 12, 644661. 34045973 10.3389/fphys.2021.644661 PMC8144521

